# Author Correction: Multivalent interactions facilitate motor-dependent protein accumulation at growing microtubule plus-ends

**DOI:** 10.1038/s41556-023-01124-w

**Published:** 2023-03-15

**Authors:** Renu Maan, Louis Reese, Vladimir A. Volkov, Matthew R. King, Eli O. van der Sluis, Nemo Andrea, Wiel H. Evers, Arjen J. Jakobi, Marileen Dogterom

**Affiliations:** 1 Department of Bionanoscience, Kavli Institute of Nanoscience, Delft University of Technology, Delft, the Netherlands; 2 Physiology Course 2017, Marine Biological Laboratory, Woods Hole, MA USA; 3 Present Address: School of Biological and Behavioural Sciences, Queen Mary University of London, London, UK; 4 Present Address: Department of Biomedical Engineering, Washington University in St. Louis, St Louis, MO USA

**Keywords:** Microtubules, Cryoelectron tomography

Correction to: *Nature Cell Biology* 10.1038/s41556-022-01037-0. Published online 19 December 2022.

In the version of this article initially published, an incorrect file for Source Data Fig. 2 was uploaded. The file has now been replaced and the error has been corrected in the HTML and PDF versions of the article.

