# Supplementary material for: Multivalent interactions facilitate motor-dependent protein accumulation at growing microtubule plus-ends
Source: Nat Cell Biol. 2022 Dec 19;25(1):68–78. doi: 10.1038/s41556-022-01037-0 (PMC9859754; doi:10.1038/s41556-022-01037-0)
Supplement: Supplementary file 1 — Reporting Summary [file 41556_2022_1037_MOESM1_ESM.pdf]

nature portfolio

Corresponding author(s): Marileen Dogterom

Last updated by author(s): 15/9/22

Reporting Summary

Nature Portfolio wishes to improve the reproducibility of the work that we publish. This form provides structure for consistency and transparency in reporting. For further information on Nature Portfolio policies, see our Editorial Policies and the Editorial Policy Checklist.

Statistics

For all statistical analyses, confirm that the following items are present in the figure legend, table legend, main text, or Methods section.

n/a	Confirmed	
☐	☒	The exact sample size (*n*) for each experimental group/condition, given as a discrete number and unit of measurement
☐	☒	A statement on whether measurements were taken from distinct samples or whether the same sample was measured repeatedly
☐	☒	The statistical test(s) used AND whether they are one- or two-sided<br>*Only common tests should be described solely by name; describe more complex techniques in the Methods section.*
☒	☐	A description of all covariates tested
☒	☐	A description of any assumptions or corrections, such as tests of normality and adjustment for multiple comparisons
☐	☒	A full description of the statistical parameters including central tendency (e.g. means) or other basic estimates (e.g. regression coefficient) AND variation (e.g. standard deviation) or associated estimates of uncertainty (e.g. confidence intervals)
☐	☒	For null hypothesis testing, the test statistic (e.g. *F*, *t*, *r*) with confidence intervals, effect sizes, degrees of freedom and *P* value noted<br>*Give P values as exact values whenever suitable.*
☒	☐	For Bayesian analysis, information on the choice of priors and Markov chain Monte Carlo settings
☒	☐	For hierarchical and complex designs, identification of the appropriate level for tests and full reporting of outcomes
☒	☐	Estimates of effect sizes (e.g. Cohen's *d*, Pearson's *r*), indicating how they were calculated

Our web collection on statistics for biologists contains articles on many of the points above.

Software and code

Policy information about availability of computer code

Data collection	Fluorescence data were recorded using Metamorph 7.8.8.0. Tomographic tilt-series were recorded using SerialEM 3.8.5.<br>The simulation code is available at https://github.com/luiree/TipPhase.
Data analysis	Fluorescence microscopy images were analyzed using Fiji 2.0<br>Tomograms were resonstructed using IMOD v. 4.9.2. Segmentation was performed using EMAN v 2,2.<br>Python scripts used for splitting of movie frames, reconstruction of even and odd tomographic volumes, training data generation, model training and denoising are available at https://github.com/NemoAndrea/cryoCARE-hpc04.

For manuscripts utilizing custom algorithms or software that are central to the research but not yet described in published literature, software must be made available to editors and reviewers. We strongly encourage code deposition in a community repository (e.g. GitHub). See the Nature Portfolio guidelines for submitting code & software for further information.

Data

Policy information about availability of data

All manuscripts must include a data availability statement. This statement should provide the following information, where applicable:

- Accession codes, unique identifiers, or web links for publicly available datasets
- A description of any restrictions on data availability
- For clinical datasets or third party data, please ensure that the statement adheres to our policy

Tomography data presented in Figure 2 are available from EMDB using the following accession codes: microtubule plus-end in presence of Tip1, Tea2 and Mal3 (EMD-14110), microtubule minus-end in presence of Tip1, Tea2 and Mal3 (EMD14111), microtubule plus-end in presence of Mal3 (EMD-1408), microtubule minus-end in presence of Mal3 (EMD-14109), microtubule plus-end in absence of additional proteins (EMD-14106), microtubule minus-end in absence of additional

proteins (EMD-14107), Tip1, Tea2 and Mal3 in presence of PEG without microtubules or tubulin (EMD-14112), and Tip1, Tea2 and Mal3 in presence of both PEG and microtubules (EMD-14182). Source Data are provided with this study. All other data are available upon request.

Field-specific reporting

Please select the one below that is the best fit for your research. If you are not sure, read the appropriate sections before making your selection.

☒ Life sciences ☐ Behavioural & social sciences ☐ Ecological, evolutionary & environmental sciences

For a reference copy of the document with all sections, see nature.com/documents/nr-reporting-summary-flat.pdf

Life sciences study design

All studies must disclose on these points even when the disclosure is negative.

Sample size	No sample size pre-determination was used. Reported data were generated from at least three independent experimental replicates.
Data exclusions	No data were excluded from analysis
Replication	Reported data were generated from at least three independent experimental replicates. All data were included in the analysis reported in the paper
Randomization	No randomization was performed as it doesn't apply to our study
Blinding	No blinding was performed as it doesn't apply to our study

Reporting for specific materials, systems and methods

We require information from authors about some types of materials, experimental systems and methods used in many studies. Here, indicate whether each material, system or method listed is relevant to your study. If you are not sure if a list item applies to your research, read the appropriate section before selecting a response.

Materials & experimental systems

n/a	Involved in the study	
☒	☐	Antibodies
☒	☐	Eukaryotic cell lines
☒	☐	Palaeontology and archaeology
☒	☐	Animals and other organisms
☒	☐	Human research participants
☒	☐	Clinical data
☒	☐	Dual use research of concern

Methods

n/a	Involved in the study	
☒	☐	ChIP-seq
☒	☐	Flow cytometry
☒	☐	MRI-based neuroimaging
